# Molecular mechanisms of ferroptosis and its roles in leukemia

**DOI:** 10.3389/fonc.2023.1308869

**Published:** 2023-12-06

**Authors:** Zhe Chen, Suying Zheng, Jiongping Han, Leihua Fu, Jiaping Fu, Zhijian Zhang, Pan Hong, Weiying Feng

**Affiliations:** Department of Hematology, Shaoxing People’s Hospital (Shaoxing Hospital, Zhejiang University School of Medicine), Shaoxing, China

**Keywords:** programmed cell death, ferroptosis, leukemia, reactive oxygen species, immunotherapy, iron metabolism

## Abstract

Cell death is a complex process required to maintain homeostasis and occurs when cells are damage or reach end of life. As research progresses, it is apparent that necrosis and apoptosis do not fully explain the whole phenomenon of cell death. Therefore, new death modalities such as autophagic cell death, and ferroptosis have been proposed. In recent years, ferroptosis, a new type of non-apoptotic cell death characterized by iron-dependent lipid peroxidation and reactive oxygen species (ROS) accumulation, has been receiving increasing attention. Ferroptosis can be involved in the pathological processes of many disorders, such as ischemia-reperfusion injury, nervous system diseases, and blood diseases. However, the specific mechanisms by which ferroptosis participates in the occurrence and development of leukemia still need to be more fully and deeply studied. In this review, we present the research progress on the mechanism of ferroptosis and its role in leukemia, to provide new theoretical basis and strategies for the diagnosis and treatment of clinical hematological diseases.

## 1 Introduction

The term “ferroptosis” was coined in 2012, when screens for small-molecule compounds capable of inhibiting the growth of RAS-mutant cancer cells were performed. In the 1950s, Harry Eagle et al. found that cysteine-deficient cells had a different pattern of cell death than those caused by other amino acid deficiencies. In the 1970s, a cysteine-dependent liver cell death involving glutathione (GSH) depletion was reported. At the same time, Shiro et al. found that alpha-tocopherol, an inhibitor of lipid peroxidation, saved cell death from GSH and cysteine deficiency. Ursini et al. isolated an enzyme named glutathione peroxidase 4 (GPX4) in 1982, which can inhibit iron-catalyzed lipid peroxidation. GPX4 protects cell death related to lipid peroxidation and oxidative stress. Dolma et al. discovered in 2003 that a small molecule compound named erastin could target the inhibition of RAS expressing tumor cells. Erastin induced death cell showed no apoptotic features and could not be inhibited by apoptosis inhibitors, suggesting new non-apoptotic cell death form. In 2012, Dixon et al. coined the term “ferroptosis” as erastin induced cell death. Ferroptosis refers to an iron-dependent form of regulatory cell death caused by lipid peroxide overload on the cell membrane. This is a new kind of cell death, which is different from the traditional forms of autophagy, apoptosis, necrosis, and other cell death. Morphologically, mitochondrial volume decreases, density increases, mitochondrial crest disappears, and lipid reactive oxygen species (ROS) increases in the cytoplasm (1). The fatal accumulation of lipid peroxides is a fundamental feature of ferroptosis and involves the confrontation between ferroptosis production and ferroptosis defense systems in cells. Ferroptosis occurs when its promotion of cellular activity significantly exceeds the antioxidant buffer provided by the ferroptosis defense system (2–4).

Ferroptosis is affected by a range of different genes including multiple cancer-related signaling pathways which have been shown to participate in ferroptosis. For example, p53 and BRCA1-related protein 1 (BAP1) induce ferroptosis in tumor cells through multiple signaling pathways, which act as a natural barrier to cancer development (5, 6). Oncogene-mediated or oncogene-signal-mediated ferroptosis avoidance contributes to tumor occurrence, progression, metastasis, and treatment resistance regulation (7, 8). Conversely, the unique metabolism of cancer cells, their high load of ROS, and their specific mutations make some of these cells inherently susceptible to ferroptosis, thus exposing therapeutic targets for certain cancer types (9–11). With the continuous development of research, ferroptosis has been confirmed to be closely related to the occurrence of tumors, respiratory system, cardiovascular system, nervous system, ischemia reperfusion injury, and other diseases. Recent studies have shown that ferroptosis also plays an important role in the development and progression of hematological diseases, especially leukemia. The present study mainly describes the role of ferroptosis in leukemia, research progress and provides new targets and new ideas for the diagnosis and treatment of leukemia.

## 2 Mechanism of ferroptosis

**Figure 1:**
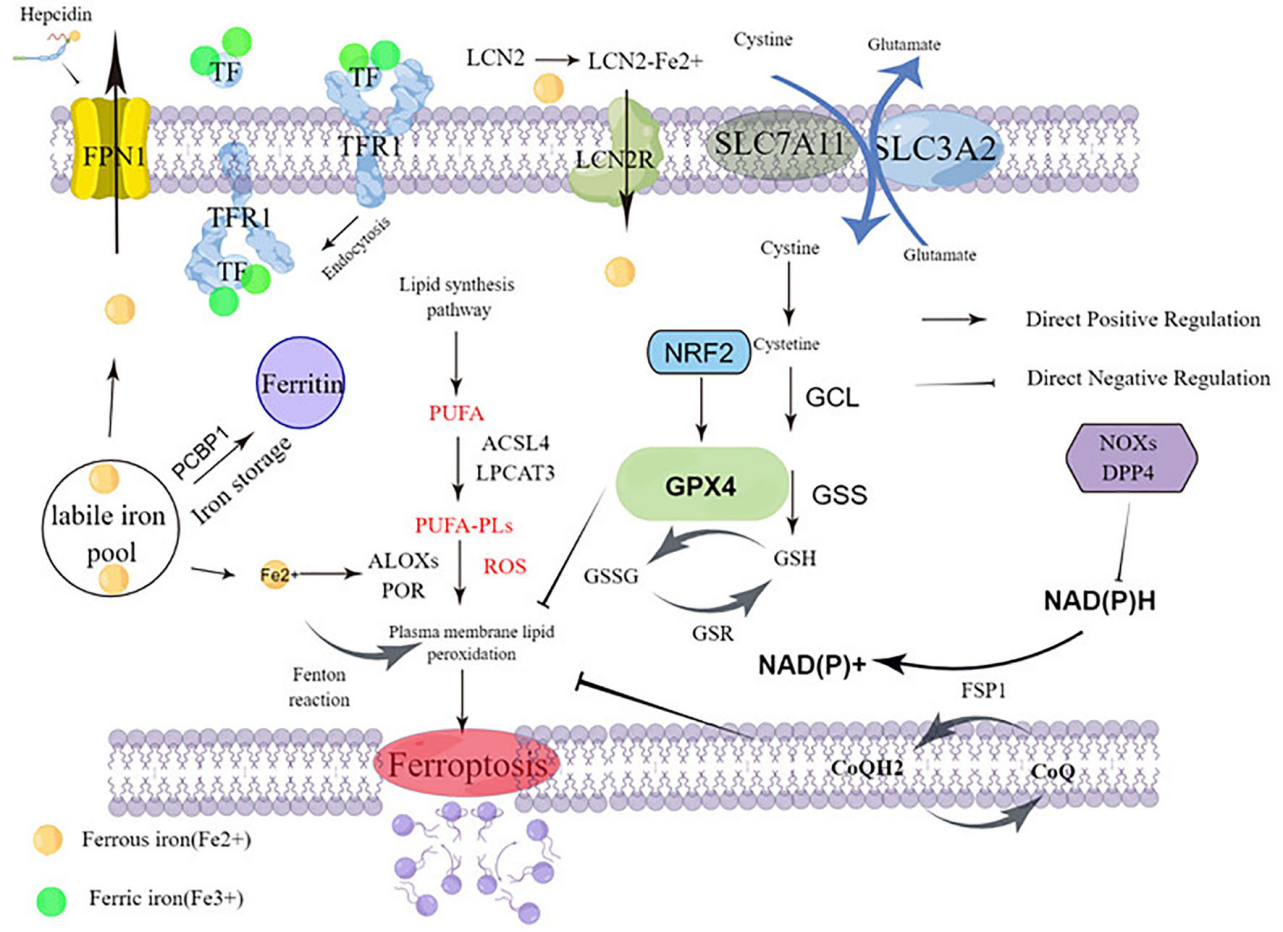
Molecular mechanisms of ferroptosis.

Ferroptosis is caused by the accumulation of lipid peroxidation, leading to the destruction of membrane structures. The prerequisite for ferroptosis is polyunsaturated fatty acids -containing phospholipids (PUFA-PLs) synthesis with peroxidation. Sensitivity to ferroptosis is regulated by several factors, including GSH and REDOX regulatory systems, such as System Xc-, GPX4 regulation, CoQ10-NAD (P) pathway, glutamine metabolic pathway, and NRF2 regulation (**Figure 1**). In this section, there are mainly describes Ferroptosis prerequisites, Ferroptosis defense mechanisms, and Upregulation of ferroptosis defenses.

### 2.1 Ferroptosis prerequisites

The crux of ferroptosis execution is PUFA-PLs synthesis with peroxidation. As outlined in this section, PUFA-PL synthesis and peroxidation, Iron metabolism, and Mitochondrial metabolism constitute the main prerequisites driving ferroptosis.

**PUFA-PL synthesis and peroxidation:** The key to triggering ferroptosis is the catalytic oxidation of phospholipids containing PUFA into polyunsaturated fatty acids, which leads to the fatal accumulation of lipid peroxides on the cell membrane and subsequent membrane rupture, resulting in ferroptosis. It is the main prerequisite of ferroptosis. Acyl-coenzyme A (CoA) synthetase long chain family member 4 (ACSL4) and lysophosphatidylcholine acyltransferase 3 (LPCAT3) are critical mediators of PUFA-PL synthesis (12, 13). ACSL4 and LPCAT3 play an important role in the biosynthesis and remodeling of phosphatidylethanolamine (PE), which can activate PUFAs and affect transmembrane properties (14). ACSL4 catalyzes the linking of free PUFAs, which include arachidonic and adrenal acid to CoA forming PUFA-CoAs such as arachidonic acid-CoA or adrenal acid -CoA. These products are then subsequently re-esterified by LPCAT3 and incorporated into PLs to form PUFA-PLs which include arachidonic acid-phosphatidylethanolamine or adrenic acid-phosphatidylethanolamine (**Figure 1**).

Acetyl-coa carboxylase (ACC) catalyzes the carboxylation of acetyl-CoA to produce malonyl CoA, which is required for synthesis of some PUFAs (15, 16). Human cytochrome P450 redox reductase (POR) mediated or arachidonic lipoxygenase (ALOXs) mediated enzymatic reactions have also been shown to promote lipid peroxidation (17, 18) (**Figure 1**). POR’s ability to promote lipid peroxidation appears to be indirect, through the production of H2O2 (18). The ALOX gene plays an important role in driving ferroptosis. The mammalian ALOX family, consisting of six members (ALOXE3, ALOX5, ALOX12, ALOX12B, ALOX15, and ALOX15B), which play a context-dependent role in driving ferroptosis. For example, spermidine/spermidine n1-acetyltransferase 1 (SAT1), a target gene for tumor protein p53 (TP53), mediates the expression of ALOX15 (but not ALOX5 and ALOX12) and is involved in TP53-mediated ferroptosis (3). Interestingly, other studies have cast doubt on the role of ALOX genes in ferroptosis (19). ALOX12 does not depend on GPX4 and ALOX15 binds to phosphatidylethanolamine binding protein 1 (PEBP1), mediating RSL3-induced ferroptosis in bronchial epithelial cells, renal epithelial cells, and neuronal cells (20).

**Iron metabolism:** Iron is a key nutrient involved in ATP production via the mitochondrial chain complex, DNA synthesis in the process of ribonucleic acid reductase, oxygen transport, antioxidant defense (peroxidase and catalase), oxygen sensitive factors such as hypoxia-inducer factor-HiIF - and proline hydroxylase, and many other enzymes. Nutrient iron exists mainly as iron ions, which can be reduced by iron reductase. Systemic iron homeostasis is maintained by a balance of iron uptake, recycling, and loss. Iron mainly comes from food intake and the elimination of aging red blood cells, existing as Fe2+ and Fe3+. Ferrous ions are internalized into intestinal cells by active transport mechanisms in the gastrointestinal tract. Iron can also be internalized into the blood through the basolateral membrane through ferroportin 1 (FPN1; the only known iron exporter), iron through the binding of ferriferous carriers to lipidin-2 (LCN2), and subsequent endocytosis returned into the cell (21, 22). However, Fe3+ combines with transferrin (TF) on the cell membrane to form TF-Fe3+, which is finally combined with transferrin receptor 1 (TFR1) and is swallowed *in vivo*. Excess iron is stored in the liver primarily through ferritin (FTH and FTL). High iron levels cause the liver system to secrete hepcidin, the most relevant regulator of iron metabolism in the system. Hepcidin is a protein of iron transport from cells which binds to ferritin transporters on iron-storage cells such as intestinal epithelial cells and macrophages. This leads to internalization and degradation of the hepcidin-transporter complex, which effectively shuts down nutrient iron uptake and iron release from internal iron stores. Expression of hepcidin is controlled by a regulatory feedback mechanism of active erythropoiesis: erythropoiet-derived erythroferone (ERFE), growth differentiation factor 11 (GDF11), growth differentiation factor 15 (GDF15), and twisted gastrin protein homology 1 (TWSG1) have been shown to affect liver hepcidin secretion. Interestingly, leukemia cells require more iron than normal cells. In particular, cancer patients require a large number of red blood cell transfusions due to normal dyserythrogenesis and anemia caused by chemotherapy, and excess iron is common in leukemia patients.

Excess iron and reactive oxygen species (ROS) catalyze production and promote malignant transformation of hematopoietic stem cells through niacinamide adenine dinucleotide phosphate oxidase (NOX) and subsequent glutathione (GSH) consumption (23).

Iron’s ability to gain and lose electrons between its oxidized Fe3+ and Fe2+ forms allow it to participate in radical generation reactions. Among these processes is the Fenton reaction, where ferrous iron contributes an electron to hydrogen peroxide to produce hydroxyl radicals that induce ROS production. Abnormal iron accumulation and subsequent excess ROS levels produce oxidative stress, induce DNA, protein, or lipid damage, and even lead to cell death. It is important to note that these oxidation actions of iron can promote the development of tumors and are thought to be necessary for the development of cancer (24). Nuclear receptor coactivator 4 (NCOA4) is the target of the ferritin transporter, which mediates ferritinophagy, a selective autophagy that degrades ferritin by lysosomes. Selective autophagy degrades ferritin. Ferritinophagy increases free iron in cells. Iron pools can be coated by lysosomes via NCOA4, and then degrade and release a large amount of Fe2+, which increases the sensitivity of cells to ferroptosis (25). Inhibition of ferritin macrophages mediated by NCOA4 increases iron storage and limits iron cell apoptosis in ferroptosis (25, 26)(**Figure 1**).

**Mitochondrial metabolism:** Overexpression of ferritin mitochondria (FTMT), an iron-storage protein in mitochondria whose primary function is to provide energy for cells through oxidative phosphorylation, is a major site of iron metabolism and ROS production which inhibits erastin-induced ferroptosis in neuroblastoma cells (27). This suggests that FTMT has a widespread anti-iron declining effect. Iron chaperone PCBP1 delivers Fe2+ to ferritin, thereby limiting ferroptosis in hepatocytes (28). The role of mitochondria in the biosynthetic pathway of cell metabolism also contributes to ferroptosis. Ferroptosis requires tricarboxylic acid (TCA) cycling (29) and various non-fusion reactions in mitochondria. These reactions may drive ferroptosis by promoting ROS, ATP, and/or PUFA-PL production (30, 31).

### 2.2 Ferroptosis defense mechanisms

The imbalance between injury and defense signals eventually leads to cell death. Ferroptosis defence mechanisms involve cellular antioxidant systems that directly neutralize lipid peroxides. As discussed below, there are mainly introduction the following two systems.

**The GPX4–GSH system:** GPX4 belongs to the GPX protein family (32, 33) and is the only GPX member capable of converting PL hydroperoxides into PL alcohols (34, 35). GPX4 is a key regulator of ferroptosis, and inhibition of its activity leads to the accumulation of lipid peroxides in cells, which signal ferroptosis in cells. Down-regulation of GPX4 increased susceptibility to ferroptosis, while up-regulation inhibited ferroptosis (36). GPX4 consists of three subtypes with different subcellular localization, namely cytoplasmic GPX4, mitochondrial GPX4, and nuclear GPX4. These isomers are encoded by the same GPX4 gene and have different transcription start sites, resulting in the n-terminal GPX4 protein of the mitochondrial or nuclear localization sequence. Only cytoplasmic GPX4 has a protective effect against ferroptosis (37). Cytoplasmic GPX4 re-expression significantly inhibited GPX4-deletion induced cell death in mouse embryonic fibroblasts. The expression or activity of GPX4 is controlled by selenium and glutathione (38, 39). Reducing glutathione (GSH), for GPX4 is a thiol-containing tripeptide derived from glycine, glutamic acid, and cysteine, of which cysteine is the rate-limiting precursor. GSH is the most abundant reducing agent in mammalian cells and is a cofactor of many enzymes. For glutathione synthesis, it is mainly formed by the redox of cysteine through the xc-/cystine/glutamate transporter (**Figure 1**).

Cystine glutamate transporter (system Xc-) is an amino acid reverse transporter widely distributed in the phospholipid bilayer and is an important part of the cellular antioxidant system. System Xc- is composed of solute carrier family 7 member 11 (SLC7A11) solute carrier family 3 member 2 (SLC3A2), an amino acid reverse transporter that can transfer cystine into cells and glutamate 1:1. Most cancer cells acquire intracellular cysteine-mediated cystine uptake (the oxydimer form of cysteine) primarily through the Xc- system, followed by reduction of cystine to cysteine in the cytoplasm (40, 41). Through the exchange of system Xc- (40), cysteine and glutamic acid are transported inside and outside the cell, and then participate in the synthesis of GSH. Inhibition of cysteine absorption can inhibit the activity of system Xc-, which can affect the synthesis of GSH and ultimately inhibit the activity of GPX, leading to the decline of cellular antioxidant capacity, lipid ROS accumulation, and inducing ferroptosis. Overexpression of apoptosis-inducing factor associated 2 (AIFM2) may eliminate GPX4 to inhibit ferroptosis (42) (**Figure 1**).

Seven members of solute vector family 11 (SLC7A11; Also called xCT) (43) is a transporter subunit in the system Xc-. The expression or activity of SLC7A11 is regulated by many factors, such as TP53 (6), NRF2 (44), BRCA1-related protein 1 (BAP1) (5), and BECN1 (45). Inhibition of SLC7A11 by small molecule compounds (such as erastin) can cause glutathione depletion to trigger ferroptosis (46). After GPX4 inactivation (10), some cancer cell lines remain resistant to ferroptosis, suggesting the presence of additional ferroptosis defense mechanisms (**Figure 1**).

**CoQH2 system:** Some recent studies suggest that ferroptosis defense systems can be divided into two main parts, GPX4 system and CoQH2 system. CoQH2 is an endogenous ferroptosis inhibitor, which has antioxidant effect in cell membrane and can reduce the oxidative damage of cell membrane. In addition to GPX4 system, DHODH/FSP1/CoQ pathway is another key inhibitory mechanism for lipid peroxidation and ferroptosis. DHODH is an enzyme involved in pyrimidine synthesis, which can reduce ubiquinone (CoQ) to ubiquinol (CoQH2) in the mitochondrial inner membrane. When GPX4 is dramatically inactivated, the flux through DHODH (3) increases significantly, leading to enhanced CoQH2 production, neutralizing lipid peroxidation, and defense against ferroptosis in mitochondria. The inactivation of mitochondrial GPX4 and DHODH releases powerful mitochondrial lipid peroxidation and triggers intense ferroptosis. Cytoplasmic GPX4 was also found to be significantly localized in the mitochondrial membrane gap (37) (**Figure 1**).

As a major suppressor of ferroptosis, FSP1 was originally described as a p53 response gene and therefore was originally called p53 response gene 3 (PRG3). FSP1, also known as AIFM2, is localized in the plasma membrane (as well as other subcellular compartments), and its plasma membrane localization appears to be necessary and sufficient to function FSP1’s role in inhibiting ferroptosis (47, 48). Doll et al. and Bersuker et al. found that FSP1 inhibits lipid peroxidation and ferroptosis by reducing CoQ (or its partially oxidized product hemihydroquinone) to CoQ2. This may directly reduce lipid radicals to terminate lipid autoxidation, or indirectly via regenerating oxidized α-tocopheryl radical (Vitamin E), a powerful natural antioxidant (47, 48). FSP1 acts as a NADPH-dependent CoQ redox enzyme, and can catalyze CoQ10 regeneration depending on NADPH, thereby improving the ability of free radical capture to protect cells, and also has a protective effect against ferroptosis caused by GPX4 deletion. This protective effect of CoQ reveals why some cells and tissues, such as highly metabolically active liver cells, contain large amounts of extracellular CoQ, which is inconsistent with its typical role in the mitochondrial electron transport chain. CoQ is synthesized mainly in the mitochondria (49), but detected in non-mitochondrial membranes, including the plasma membrane (50). Unanswered questions remain about the potential role of other CoQH2 producing mitochondrial enzymes in the regulation of ferroptosis.

### 2.3 Upregulation of ferroptosis defenses

Despite the prevailing importance of GPX4 and CoQH2 for limiting ferroptosis, both the signal pathways and the tumor microenvironment influence the function of ferroptosis in tumorigenesis and tumor therapy.This section focuses on the role of these two pathways that inhibit ferroptosis.

**Hippo–YAP signaling in ferroptosis:** The Hippo -Yap pathway is involved in a variety of biological functions, including cell proliferation and organ size control (51). Wu et al. demonstrated the role of intercellular interactions and intracellular NF2-YAP signaling in dictating ferroptosis, which can promote the survival of GPX4 knockout cells (9). Because YAP targets include several regulators of ferroptosis, including transferrin receptors ACSL4, TFR1, and possibly other genes, susceptibility to ferroptosis depends on Hippo pathway activity, with increased susceptibility in response to Hippo inhibition and YAP activation (9). Yang et al. found that in renal cell carcinoma (RCC), Transcription Regulator 1 (TAZ) is abundantly expressed and regulates ferroptosis through Epithelial Membrane Protein 1 (EMP1)-NOX4 (52).

**Nuclear factor E2 erythroid 2-like-2 (NRF2)**: The NRF2 transcription pathway can up-regulate the expression of antioxidant genes or cell protective genes in various oxidative stress processes. As a major regulator of antioxidant defense, transcription factor NRF2 (53, 54) controls the transcription of many genes involved in GPX4-GSH-mediated ferroptosis defense. Sun et al. report that NRF2 plays a central role in protecting hepatocellular carcinoma (HCC) cells against ferroptosis, and NRF2 signaling is up-regulated in many human cancer types (55) (**Figure 1**).

**Ferroptosis in the tumor microenvironment:** Recent studies have also shown that the tumor microenvironment (TME), which is a multicellular environment, includes the extracellular matrix, immune cells, blood vessels, tumor cells, and other cells. In particular, immune cells determine whether ferroptosis in tumor cells will occur. CD8+ cytotoxic T cells are the main agents of anti-tumor immunity in the TME (56), secreting interferon-γ (IFN-γ) and subsequently inhibiting cystine uptake by cancer cells via down-regulation of SLC7A11 expression, thereby increasing lipid peroxidation and ferroptosis in tumors. Ferroptotic cancer cells can release several immunostimulatory signals, such as high mobility group box 1 (HMGB1) (57), calreticulin (58), ATP (59), and phosphatidylethanolamine (60). These factors can promote dendritic cell maturation, increasing the efficiency of macrophages in the phagocytosis of ferroptotic cancer cells, and further enhance the infiltration of CD8+ T cells into tumors.

Immunotherapy, combined with induction of ferroptosis, is a promising therapeutic approach. Drijvers et al. (61) found that Acyl-CoA synthetase ACSL4 mediates GPX4 inhibitor-induced sensitivity changes and ferroptosis in activated CD8+ T cells. CD8+ T cells can inhibit tumor cells by inducing iron decay and pyrosis (62, 63).

## 3 The role of ferroptosis in leukemia

Leukemia comprises a group of heterogeneous hematopoietic stem/progenitor cell malignancies characterized by abnormal proliferation of primitive cells in the bone marrow that interfere with normal blood cell production. Its occurrence involves multiple gene changes including the transferrin receptor 1 gene, the hemochromatosis (HFE) gene, and several genes related to iron metabolism.

At present, chemotherapy, immunotherapy, and hematopoietic stem cell transplantation (HSCT) are still the main treatments for leukemia. Despite advances in treatment, the results remain disheartening. Relapse or refractory disease and resistance to chemotherapy are the main reasons for treatment failure. Overcoming drug resistance is a major challenge in cancer treatment. Combination therapy prevents drug resistance by combining drugs with different targets, modes of action, and distribution of side effects in the body to reduce toxicity (64).

As a newly discovered programmed cell death mode, ferroptosis is regulated by multiple pathways such as lipid metabolism, mitochondrial metabolism and iron metabolism. Through this new death mode, it provides a new idea for improving the prognosis of leukemia patients. However, leukemia cells seem to be able to escape oxidative stress and reduce ferroptosis through some mechanisms.

### 3.1 Acute myeloid leukemia

Acute myeloid leukemia (AML) is a clonal hematopoietic disease caused by a variety of genetic and epigenetic impairments, characterized by impaired differentiation and uncontrolled proliferation, with varying prognoses (65). The incidence of AML increases with age, with a mortality rate of over 90% (66) at diagnosis after age 65. Ferroptosis provides a new idea for the treatment of AML, and a variety of drugs have been shown to induce ferroptosis.

Sorafenib has been approved as a tyrosine kinase inhibitor for the treatment of liver, kidney, and thyroid cancers for more than 15 years, and has recently been shown to be effective in AML patients with FLT3-ITD mutations (67). Concurrently, it also inhibits system Xc- and thus induces ferroptosis (46). Imatinib Mesylate (IMA) (68)down-regulates the expression of NRF2 and up-regulates the expression of p53 and TFR. These results provided compelling evidence that ferroptosis participates in IMA-induced cardiotoxicity. Ferroptosis could be regarded as a target to protect against cardiotoxicity in IMA-exposed patients.

APR-246 (69) is a novel drug for the treatment of TP53-mutant AML. Its main mechanism of action is to promote the binding of p53 mutants to DNA targets to reactivate the transcriptional activity of p53 and exert tumor inhibitory effects. APR-246 (70) increases oxidative stress by depleting GSH and inhibiting thioredoxin reductase, leading to the accumulation of ROS and further promoting tumor cell death. Birsen et al. (71) found that the observed early p53-independent cell death induced by APR-246 is ferroptosis.

NEAT1 (72) is bound to cytoplasmic disheveled 2 (DVL2) and tripartite motif containing 56 (TRIM56), which promotes the degradation of DVL2 and inhibits Wnt signaling, inhibiting the self-renewal of AML stem cells. Zhang et al. (73) found that ferroptosis inducers erastin and RSL3 increased NEAT1 expression by promoting the binding of p53 to the NEAT1 promoter. Induced NEAT1 promoted the expression of MIOX by competitively binding to miR-362-3p. MIOX increased ROS production and decreased the intracellular levels of NADPH and GSH, resulting in enhanced erastin- and RSL3-induced ferroptosis.

Aldo-keto reductase family 1 member C2 (AKR1C2), suppressor of cytokine signaling1 (SOCS1) (74), dipetidyl peptidase-4 (DPP4) (75), and human immunodeficiency virus type I enhancer-binding protein zinc finger 3(HIVEP3) (76) can be used as predictive adverse prognosis. Long non-coding RNAs (lncrnas) (77) associated with ferroptosis have also been shown to accurately predict the prognosis of AML and optimize treatment strategies for AML.

Acetaldehyde dehydrogenase 3a2 (Aldh3a2) (78) is l-gmp dependent and not seen in n-gmp. It protects AML cells from oxidative cell death, and Aldh3a2 inhibition improves leukemia outcomes *in vivo* without compromising normal hematopoiesis. Aldh3a2 inhibition combined with ferroptosis inducer or standard AML induction chemotherapy deserves further consideration as a cancer treatment.

High mobility base Box 1 (HMGB1) (79) is a transcription factor involved in the process of chromatin remodeling, DNA recombination, and repair. HMGB1 is found in the cytoplasm and, via translocation, is expressed on the cell surface membrane or diffuse in the extracellular space. This can be caused by various cellular stressors, causing HMGB1 to migrate from the nucleus to the cytoplasm in response to erastin in HL-60/NRASQ61L cell lines and acts as a positive regulator of ferroptosis, possibly enhancing resistance to anticancer therapy.

At present, a variety of drugs have been confirmed to promote or inhibit ferroptosis in AML cells, but there is still a lack of large-scale studies, and further research is still needed to support the development. There are still many challenges in the clinical application of ferroptosis in the treatment of leukemia.

### 3.2 Acute lymphoblastic leukemia

Acute lymphoblastic leukemia (ALL) is a common malignant disease of the blood system, which manifests as abnormal clonal proliferation of naive or immature T and B lymphocytes. These cells will infiltrate bone marrow, blood, or other tissues and organs, causing abnormal hematopoietic function of bone marrow and immune dysfunction. Vincristine (VCR) is often used as a treatment for ALL (80). Studies have found that VCR promotes ferroptosis by enhancing the expression of lncRNA LINC00618 and inhibiting the transcription of SLC7A11, suggesting that ferroptosis is involved in the mechanism of action of VCR.

RSL3 is an inducer of ferroptosis that binds and inactivates GPX4, mediating ferroptosis regulated by GPX4 (81). Probst et al. treated ALL cell lines with RSL3 causing lipid peroxidation, ROS production, and cell death (82). Hydnocarpin D (HD) can trigger ferroptosis through the accumulation of lipid ROS and the reduction of GSH and GPX4, while the inhibition of autophagy prevents the ferroptosis (83). PAQR3 (progestin and adipoQ receptor family member 3) is involved in the occurrence of many tumors as a tumor suppressor and can inhibit the proliferation of human leukemia cells and induce cell apoptosis (84). Jin et al. found that PAQR3 inhibits cell proliferation and aggravates ferroptosis in ALL by regulating the stability of NRF2. Hong et al. (85, 86) demonstrated the critical role of ferroptosis in Philadelphia chromosome negative (Ph-neg) B-ALL patients, with sorafenib potentially improving survival in high-risk Ph-neg B-ALL patients.

Artesunate (ART), a semi-synthetic water-soluble derivative of Artemisia annua L., is a natural product extracted from artemisia annua L. Apoptosis induced by ART corresponded to the activation of caspase-8/9/3. The expression of Bcl-xL, Bcl-2, myeloid leukemia-1, survivin, X-linked apoptosis inhibitor protein, and apoptosis inhibitor 1/2 were decreased, with increased expression of Bak. ART increased the activation of intracellular ROS and DNA damage marker gamma-H2Ax. In the ATLL mouse model, intraperitoneal injection of ART reduced tumor burden (87).

Poricoic acid A (PAA) (88) strongly reduced the cell viability of T-ALL cell lines. Mitochondrial dysfunction was also elevated by PAA, along with enhanced cellular reactive oxygen species (ROS) production. PAA treatments provoked ferroptosis in T-ALL cells with reduced glutathione (GSH) levels and elevated malonaldehyde (MDA) contents. As a new mode of regulatory cell death, amplification of ferroptosis effect may be a new idea for drug development and disease treatment.

### 3.3 Chronic lymphocytic leukemia

Chronic lymphocytic leukemia (CLL) is a disease with different genetic characteristics and treatment responses. CLL is characterized by the cloning and proliferation of mature CD5-positive B cells in the blood, bone marrow, lymph nodes, and spleen, resulting in immune system decline, organ dysfunction, and slow progressive systemic failure and depletion.

Ferroptosis is less well studied in the CLL field. SLC7A11 is the main functional subunit of system Xc- to transport cystine into cells to synthesize GSH. Inhibition of SLC7A11 expression can induce ferroptosis. The expression of SLC7A11 is low in CLL compared to the high expression level of SLC7A11 in other systemic solid tumors. This will lead to an increase in intracellular ROS, and as CLL cells are more prone to oxidative stress, they may be sensitive to ferroptosis inducers.

Ferroptosis is an autophagy-dependent form of cell death. BECN1 affects the occurrence and progression of autophagy, and its repeated allelic deletion and expression variation have been reported in tumors (89). Gong et al. (90) proposed a novel FPS model for prognostic prediction of CLL and established nine ferroptosis genes associated with CLL prognosis.

### 3.4 Chronic myelogenous leukemia

Chronic myelogenous leukemia (CML) is a hematopoietic malignancy caused by reciprocal translocation of Philadelphia chromosomes 9 and 22. Ferroptosis has been less studied in the field of CML. Cysteine metabolism plays a key role in cancer cell survival in the study of CML related fields. Cysteine deficiency has been reported to inhibit tumor growth and induce ferroptosis in pancreatic cancer cells. It has also been reported that cysteine depletion can induce ferroptosis in CML cells *in vitro*, and thioredoxin reductase 1 (TXNRD1) (91), which is related to cell redox metabolism, is a key factor regulating ferroptosis.

## 4 Conclusion and outlook

Ferroptosis, as a newly discovered form of programmed cell death, has a broad prospect in tumor therapy. We have systematically and comprehensively illustrated the relationship between ferroptosis and leukemia, and found that ferroptosis plays an important role in disease progression. PUFA-PL synthesis and peroxidation, intracellular ROS levels, and homeostasis of various metabolic pathways can affect cell sensitivity to ferroptosis, thus inducing ferroptosis in blood cells. Iron accumulation and lipid peroxidation may be considered as intermediate events, but they are not the final executors of ferroptosis. Leukemia cells seem to escape oxidative stress through certain mechanisms, such as the upregulation of ferroptosis defenses and ferroptosis defense mechanisms, which reduce the occurrence of ferroptosis. However, the study of ferroptosis in hematological diseases is still in the early stage, and its specific mechanism needs to be further studied. At present, most studies affect the activity of antioxidants such as GPX4 through exogenous ferroptosis inducers, causing the accumulation of ROS and thus promoting ferroptosis. There are few large studies on ferroptosis inducers in the treatment of leukemia. The pathogenesis of leukemia is complex, and often involves multiple pathways and targets. The previous multi-drug combination chemotherapy with cytotoxic drugs could easily cause serious adverse consequences such as bone marrow suppression and immune destruction. Novel target inhibitors related to ferroptosis, or their combination with existing cytotoxic agents, may further enhance the efficacy of existing single agents, delay drug resistance and improve prognosis. Although the treatment of leukemia with ferroptosis inducers is not mature at present, but it still has high research value.

In summary, we are currently in the middle of an important phase in the development of ferroptosis research. The occurrence and development of ferroptosis, its transcriptional regulation mechanism, and the development of effective regulatory targeted drugs are of utmost importance, providing a new direction for clinical diagnosis and treatment of blood diseases. New treatments based on ferroptosis will be developed and put into clinical use soon, guided by specific biomarkers and a precise assessment of a patient’s background.

## Author contributions

ZC: Writing – review & editing. SZ: Data curation, Writing – original draft. JH: Data curation, Writing – original draft. LF: Writing – original draft. JF: Funding acquisition, Writing – review & editing. ZZ: Methodology, Writing – review & editing. PH: Writing – original draft. WF: Writing – review & editing.
